# Navigator‐based reacquisition and estimation of motion‐corrupted data: Application to multi‐echo spin echo for carotid wall MRI

**DOI:** 10.1002/mrm.28063

**Published:** 2019-11-07

**Authors:** Robert Frost, Luca Biasiolli, Linqing Li, Katherine Hurst, Mohammad Alkhalil, Robin P. Choudhury, Matthew D. Robson, Aaron T. Hess, Peter Jezzard

**Affiliations:** ^1^ Wellcome Centre for Integrative Neuroimaging FMRIB Division Nuffield Department of Clinical Neurosciences University of Oxford Oxford United Kingdom; ^2^ Athinoula A. Martinos Center for Biomedical Imaging Massachusetts General Hospital Charlestown Massachusetts; ^3^ Department of Radiology Harvard Medical School Boston Massachusetts; ^4^ Oxford Centre for Clinical Magnetic Resonance Research Division of Cardiovascular Medicine Radcliffe Department of Medicine University of Oxford Oxford United Kingdom; ^5^ Acute Vascular Imaging Centre Division of Cardiovascular Medicine Radcliffe Department of Medicine University of Oxford Oxford United Kingdom; ^6^ Laboratory of Brain and Cognition National Institute of Mental Health Bethesda Maryland; ^7^ Nuffield Department of Surgical Sciences University of Oxford Oxford United Kingdom

**Keywords:** atherosclerotic plaque imaging, data estimation motion correction, MR motion navigator, navigator‐based reacquisition, parallel imaging, vessel‐wall imaging

## Abstract

**Purpose:**

To assess whether artifacts in multi‐slice multi‐echo spin echo neck imaging, thought to be caused by brief motion events such as swallowing, can be corrected by reacquiring corrupted central k‐space data and estimating the remainder with parallel imaging.

**Methods:**

A single phase‐encode line (*k_y_* = 0, phase‐encode direction anteroposterior) navigator echo was used to identify motion‐corrupted data and guide the online reacquisition. If motion corruption was detected in the 7 central k‐space lines, they were replaced with reacquired data. Subsequently, GRAPPA reconstruction was trained on the updated central portion of k‐space and then used to estimate the remaining motion‐corrupted k‐space data from surrounding uncorrupted data. Similar compressed sensing‐based approaches have been used previously to compensate for respiration in cardiac imaging. The g‐factor noise amplification was calculated for the parallel imaging reconstruction of data acquired with a 10‐channel neck coil. The method was assessed in scans with 9 volunteers and 12 patients.

**Results:**

The g‐factor analysis showed that GRAPPA reconstruction of 2 adjacent motion‐corrupted lines causes high noise amplification; therefore, the number of 2‐line estimations should be limited. In volunteer scans, median ghosting reduction of 24% was achieved with 2 adjacent motion‐corrupted lines correction, and image quality was improved in 2 patient scans that had motion corruption close to the center of k‐space.

**Conclusion:**

Motion‐corrupted echo‐trains can be identified with a navigator echo. Combined reacquisition and parallel imaging estimation reduced motion artifacts in multi‐slice MESE when there were brief motion events, especially when motion corruption was close to the center of k‐space.

## 1 INTRODUCTION

MRI of the carotid arteries can detect atherosclerotic plaques at risk of rupturing and causing acute ischemic stroke.^1^ Multi‐contrast methods have been used for this purpose^2^, ^3^; more recently, quantitative T_2_ mapping with a multi‐echo spin echo (MESE) sequence has emerged as a promising technique.^4^, ^5^, ^6^, ^7^ However, segmented sequences generally have high sensitivity to movement of the neck, including unavoidable breathing, arterial pulsation, swallowing, and coughing, in addition to drift (e.g., muscle relaxation or cushion compression) and other bulk motion. Pulsation of vessels can cause relatively small carotid artery displacements (~0.5 mm),^8^, ^9^ whereas motion due to swallowing in the head–feet, left–right, and anteroposterior directions can be on the order of a few mm, thus larger‐than‐normal wall thickness and in‐plane spatial resolution.^10^, ^11^ 3D sequences have been found to be more sensitive to motion than 2D multi‐slice sequences,^12^, ^13^ and the highly segmented MESE acquisition with each slice acquired over ~4 min is also susceptible to motion.

The severity of the motion artifacts depends on the particular k‐space acquisition schedule and relative timing of movement; however, problems often manifest as ghosting, blurring, or signal loss. Movements between k‐space lines during image acquisition cause the following: 1) corruption of k‐space lines that coincide with motion, and 2) data inconsistency before/after movement. Prospective corrections, which apply rigid‐body updates to the FOV while tracking subject movement, and which have been successfully deployed in neuroimaging,^14^, ^15^, ^16^ can correct the data inconsistency problem and also aid reacquisition of corrupted data; the object–FOV relationship can be corrected to ensure that reacquired data are consistent.^17^, ^18^, ^19^ For neck imaging, movement is generally nonrigid body; therefore, prospective translations and rotations of the FOV cannot maintain a consistent view of the moving neck during data acquisition.

Previous compensation strategies for vessel wall imaging have focused on avoiding or reacquiring corrupted data through methods such as 1D tracking of the epiglottis, self‐gating, or reacquisition based on free induction decay navigators.^20^, ^21^, ^22^, ^23^, ^24^ In other settings, parallel imaging methods have been used to detect motion corruption and enable application of corrections of varying degrees,^25^, ^26^, ^27^, ^28^, ^29^ and they also have been combined with “floating” navigators (an off‐center phase‐encode line) used to estimate translation and rotation.^30^

The goal of this study was to investigate to what extent artifacts thought to be caused by swallowing events can be corrected by detecting them and then reacquiring corrupted central k‐space data and estimating the remainder with parallel imaging.^31^, ^32^ A related approach has been used previously to reduce artifacts caused by respiratory motion in cardiac imaging for which a substantial fraction (~50%‐80%) of k‐space is discarded by gating.^33^, ^34^ Moghari et al. found that after rejecting data corrupted by respiratory motion, the data was randomly undersampled. Therefore, compressed sensing was used to recover the corrupted data. Also, gating was used to ensure a motion‐free central k‐space region. We have developed a similar strategy for neck imaging with MESE. For our study, fully sampled data are acquired to obtain high‐resolution images of the small vessel walls with sufficient SNR. Furthermore, often only a small fraction of the data are motion‐corrupted; thus, we propose to use conventional parallel imaging to estimate corrupted data.

A *k_y_* = 0 navigator echo was added to the end of each MESE readout to detect motion corruption in spin echo trains.^35^, ^36^ Corrupted k‐space lines were identified in real time so that they could be reacquired at the end of the scan.^37^, ^38^ In reconstruction, we used a hybrid *MoCo* approach of re‐acquiring corrupted auto‐calibration lines and estimating corrupted lines using parallel imaging algorithms.^33^, ^34^, ^39^ Reacquisition may offer robustness to the cases when crucial central k‐space data are affected by motion and parallel imaging reconstruction cannot be calibrated (see Supporting Information Figure S1). In these cases, corrupted data were discarded; next, the central k‐space data were replaced with reacquisitions; and finally, parallel imaging reconstructions trained on the central k‐space data were used to estimate the remaining corrupted data. In motion tests with healthy volunteers, the MoCo strategy was compared with replacement with reacquisitions, the original motion‐corrupted images, and gold standard scans without motion. In these volunteer comparisons, ghosting artifact, T_2_ values, image sharpness, and vessel–lumen contrast to noise in MoCo images were assessed. Finally, the efficacy of the proposed acquisition and reconstruction for detecting and correcting motion was assessed in 12 patients with carotid atherosclerosis.

## 2 METHODS

### 2.1 Navigator‐based motion detection and reacquisition

**Figure 1:**
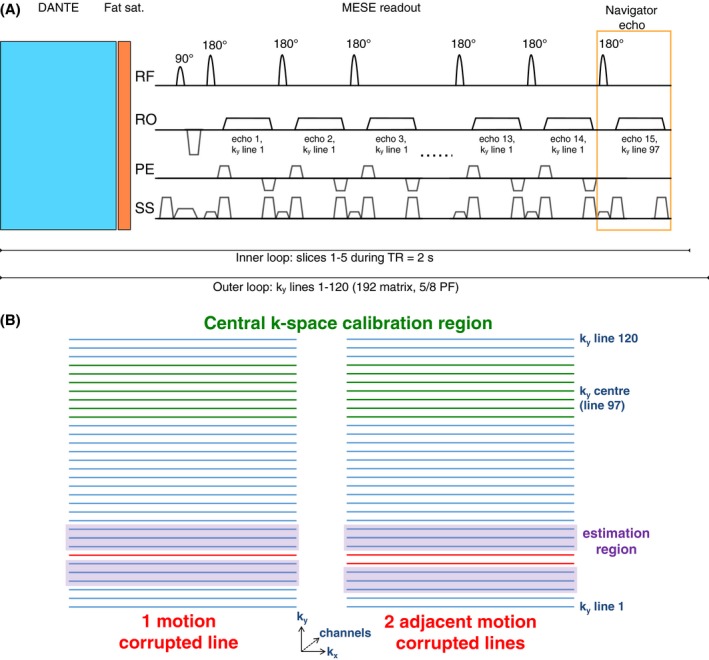
(A) Pulse sequence diagram for the readout of a single k‐space line of a single slice. During each TR, this sequence block was repeated for all slices. The DANTE module was used for black‐blood preparation. The navigator acquisition of the *k_y_* = 0 line (# 97) was added at the end of the echo train, which originally consisted of 14 echoes. (B) Schematic of the reconstruction of a single AMCL (1‐AMCL) and a pair of lines (2‐AMCL), shown in red, from surrounding data in the estimation region (purple). The GRAPPA reconstruction is calibrated using the high SNR central k‐space region (green). The k‐space data from the other channels are not shown but are indicated by the dashed axis. Note that the schematic is for a partial Fourier acquisition; thus, the phase‐encode sampling is asymmetric about the center of k‐space. AMCL, adjacent motion‐corrupted lines; DANTE, delay alternating with nutation for tailored excitation

The multi‐slice MESE implementation used in this study is shown in Figure 1A. The acquisition block for a particular slice and anteroposterior phase‐encoding (*k_y_*) line consisted of 14 spin echoes with different TEs.^4^ All slices were acquired during the 2 s TR, and the *k_y_* index was incremented in successive TRs. To avoid interfering with the T_2_ mapping, 1 additional navigator echo was added to the end of the echo train, increasing the duration of each slice acquisition block by 9.1 ms. The navigator echo is a readout without phase‐encoding (anteroposterior *k_y_* = 0, equivalent to the 97th phase‐encoding line of a 192 matrix). This resulted in no increase in TR required for an acquisition with 5 slices. The TE of the navigator acquisition was 136.5 ms. The sum of the magnitude signal across all channels in each navigator was used as a slice score *S_sl_* (*k_y_*) for each echo train:

$$S_{\mathrm{sl}}\left(k_{y}\right)=\sum_{c}\sum_{k_{x}}\left|N_{\mathrm{sl}}\left(k_{x},\;k_{y},\;c\right)\right|,$$

where *sl* refers to the slice number, and the complex navigator signal for slice *sl* and line *k_y_* at k‐space location *k_x_* in channel *c* is given by *N_sl_* (*k_x_*, *k_y_*, *c*). This navigator signal will be reduced should any motion occur during the echo train due to dephasing of spin echoes. In addition, the signal magnitude will change^36^ should the total signal in the slice change and if excited tissue is not refocused, for example, if there through‐plane movement of the tongue and throat during swallowing. The signal from all coils was summed to detect motion anywhere in the coil's sensitive region, that is, the neck. Assuming that the neck returns to a similar position after swallowing, corrupted data can be replaced or estimated.

TR periods were chosen for reacquisition after ranking based on the sum of slice scores within each TR block (5 slices in this study); we call this the *quality score* $S_{\mathrm{TR}}=\sum_{\mathrm{sl}}S_{\mathrm{sl}}\left(k_{y}\right)$. The lowest quality scores were reacquired at the end of the scan, with the number of reacquisitions specified by the scanner operator in advance.

### 2.2 Motion experiments

Nine healthy volunteers (mean ± SD in age and weight: 33.2 ± 7.0 years; 78 ± 5 kg) were scanned under a technical development ethics agreement on a Siemens (Erlangen, Germany) Verio 3T scanner. The following MESE acquisition parameters were used: 14 echoes (TE = 9.1‐127.4 ms), TR = 2 s, FOV = 128 × 128 mm^2^, matrix size = 192 × 192, 5/8 partial Fourier (120 acquired phase‐encoding lines of which line 97 was the central k‐space line), and five 2 mm slices (100% slice gap) acquired in an interleaved order. A 60 ms delay alternating with nutation for tailored excitation (DANTE) preparation before each readout module was used for flowing spin suppression, which has been shown to offer increased vessel wall to lumen contrast‐to‐noise compared with conventional double inversion‐recovery and motion‐sensitive driven equilibrium black‐blood techniques.^40^, ^41^ The following parameters were used for the DANTE module: 18 mT/m slice gradient amplitude, 120 pulses, 60 μs nonselective RF pulse duration, 8° flip angle, 500 μs between RF pulses, and 340 μs gradient duration. Sixteen reacquisition TRs were acquired (for comparison of reacquisition replacement vs. data estimation), resulting in a total scan time of 4:36 min (reduced to 4:14 min when using only 5 central k‐space weighted reacquisitions). Images were reconstructed offline in MatLab R2017a (MathWorks, Natick, MA). Projection onto convex sets (POCS) partial Fourier reconstruction^42^ was used after replacement of data with reacquired or estimated data (see below). Data were acquired with a purpose‐built 10‐channel phased‐array carotid coil (PulseTeq, Surrey, UK).

For each volunteer, a scan without intentional motion and 2 scans with 4 or 5 swallowing movements (referred to as scans A and B in Figures, e.g., “subject 3A”) were acquired with the modified MESE sequence. The intentional motion period started at 1:30 min into the scan and lasted until the end, during which time volunteers were instructed to swallow approximately every 30 to 45 s. Therefore, *k_y_* lines in the range 40 to 120 (192 phase‐encode matrix size with 5/8 partial Fourier) were affected by motion.

### 2.3 Estimation of motion‐corrupted data

Drift in the quality scores during the scan was accounted for by subtracting the local median score using a sliding window of width 10. The motion‐corrupted lines were then identified in the drift‐adjusted scores by finding scores lower than the local median (window width = 25) by 3 SDs (see hampel.m function in MatLab R2017a [MathWorks]).

For a chosen number of corrupted lines, the following reconstruction procedure was followed. First, to ensure that the crucial part of the calibration region was not motion‐corrupted, motion‐corrupted data in the 7 lines at the center of k‐space were replaced by reacquisitions provided that the reacquired data had higher quality scores. Then, GRAPPA kernels^32^ were trained from the data in the calibration region to estimate single corrupted lines and pairs of corrupted lines from the surrounding data, as shown in Figure 1B. The 1‐ and 2‐AMCL (adjacent motion‐corrupted lines) GRAPPA reconstruction of single lines and pairs of lines, respectively, corresponds to R = 2 and 3 undersampling in conventional parallel imaging acceleration. However, the source points for reconstruction can be tighter around the particular line(s) than in conventional GRAPPA because the overall acquisition is not undersampled (save for the missing lines due to motion corruption). The phase‐encode lines were ranked by their quality scores, and the lines with the lowest (worst) scores were estimated first. A maximum of 2‐AMCL GRAPPA was used; thus, for example, in the case of >2 adjacent corrupted lines, only the 2 lines with the lowest quality scores would be estimated.

Corrupted lines were also estimated with the iterative self‐consistent parallel imaging reconstruction algorithm (SPRIRiT)^43^ for comparison (after the same replacement of corrupted calibration region data) using code available at https://people.eecs.berkeley.edu/~mlustig/Software.html (version 0.3). In healthy volunteer scans with 16 reacquisitions, images were also reconstructed by replacing corrupted data with reacquisitions (if the quality score of the reacquired line was higher than the original quality score) for comparison.

Image quality and performance with high numbers of estimated lines were assessed for iterative parallel imaging with l2‐norm (*λ* = 0.1) and total variation (*λ* = 0.005) regularization. Images were reconstructed with the BART toolbox (version 0.4.03).^44^ Estimation of signal parameters via rotational invariance technique was used to estimate coil sensitivities (ESPIRiT)^45^ used in the iterative reconstructions.

The reduction in SNR associated with the GRAPPA reconstruction was assessed using the procedure described in Breuer et al. to estimate the resulting g‐factor after application of a combination of different GRAPPA kernels.^46^ This corresponds to a weighted sum of the different g‐factor contributions of the different kernels.^31^

### 2.4 Postprocessing and data analysis

The scans with swallowing motion corruption and the original image reconstruction are referred to as *SWL*. The same motion‐corrupted data, with replacement of calibration data followed by GRAPPA reconstruction, are referred to as *MoCo*. Unless otherwise stated, MoCo refers to 2‐AMCL GRAPPA reconstruction. The reconstructions only using replacement of data with reacquisitions are referred to as *Reacq*. The gold standard scans without intentional motion and with original reconstruction are referred to as *Still*.

Ghosting in the motion‐corrupted MESE images was assessed by first isolating the ghosting level (by removing the background noise contribution) and then normalizing to the original image reconstruction (no data estimation or reacquisition replacement), as demonstrated in Supporting Information Figure S2. To isolate the ghost level in each image reconstruction, the median intensity in a background ROI of the original image reconstruction was subtracted from the median intensity in a ghosted region of interest. An example of the ghost and background regions of interest defined in each image is shown in Supporting Information Figure S2A.

To study the effect of motion correction on carotid arteries, the lumen and external wall boundary were segmented, and T_2_ maps were generated following a published procedure.^4^ The difference between corrected and original images was measured for T_2_, wall/lumen contrast, and vessel edge sharpness using image edge profile acutance (IEPA).^47^ Wall/lumen contrast‐to‐noise ratio (CNR) and image edge profile acutance were measured for every vessel wall image at each of the 14 TEs (n = 9 volunteers × 2 carotid arteries × 5 slices). We used the 2‐sample *t* test (with equal variance) between Still and SWL or between Still and MoCo (2‐AMCL GRAPPA) metrics because they were acquired at different times and we could not assume that slices were at the same locations. However, we used the paired *t* test between SWL and MoCo metrics because the acquired k‐space data were the same (except for reacquired and estimated lines). T_2_ values were estimated voxel‐wise in the vessel wall (n ≈ 9000 for 2 carotid arteries with 5 slices each in 9 volunteers), and we used the 2‐sample *t* test (with equal variance) for all the comparisons between Still, SWL, and MoCo.

### 2.5 Patient scans

Twelve patients with carotid atherosclerosis (mean ± SD in age and weight: 72.3 ± 9.4 years; 80.6 ± 11.7 kg) were scanned using the navigated MESE sequence. A shorter reacquisition period of 5 TRs, weighted toward central k‐space lines, was used. The weighting function is shown in Supporting Information Figure S3.

## 3 RESULTS

**Figure 2:**
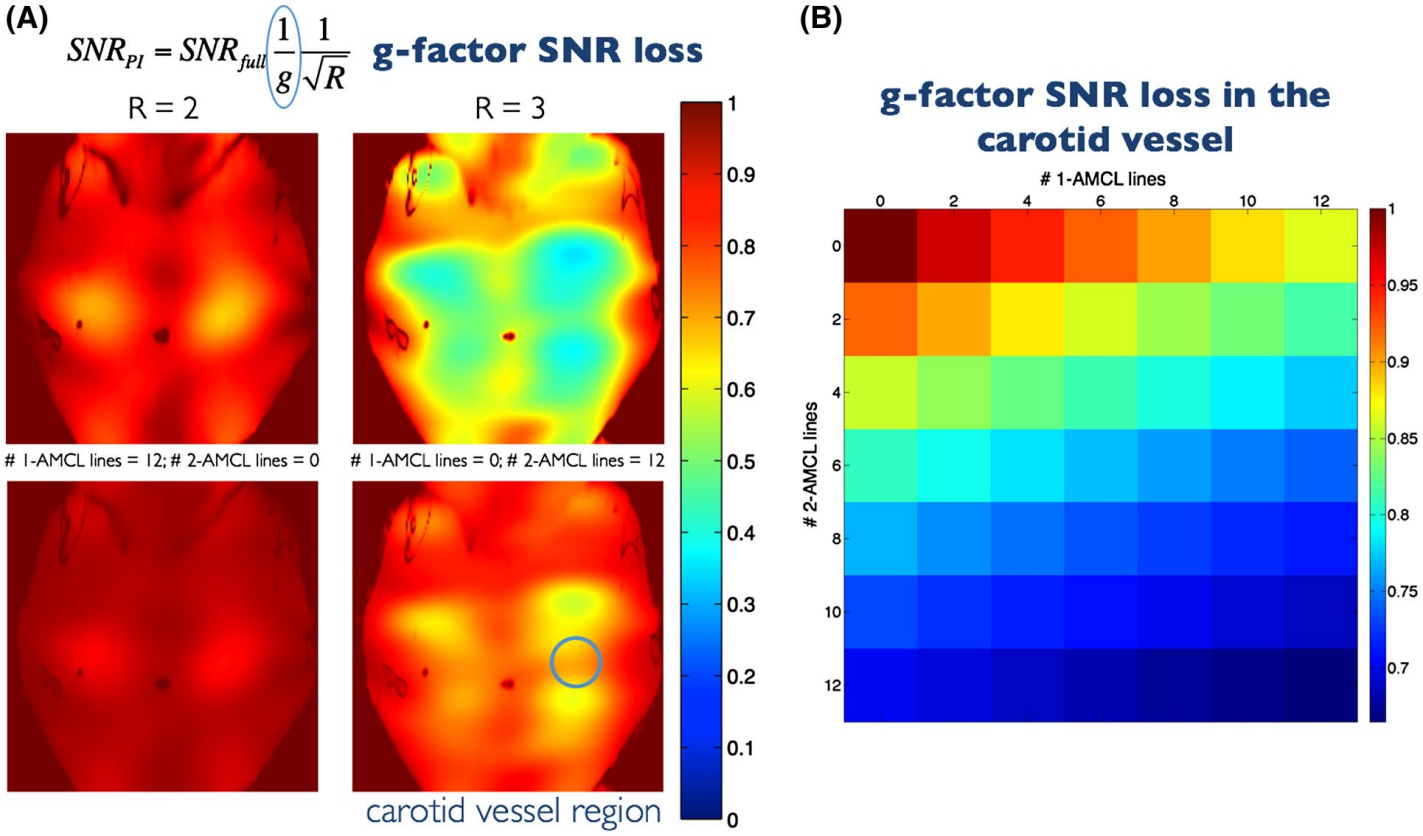
Estimated SNR loss due to the g‐factor of various reconstructions. (A) Top row: Retained SNR (1/g) of conventional R = 2 and R = 3 GRAPPA acquisitions (half and a third of the fully sampled number of lines acquired). Bottom row: Retained SNR (1/g) when estimating 12 lines with 1‐AMCL and 2‐AMCL GRAPPA acquisitions. (B) Retained SNR in an ROI around the vessel wall for various combinations of 1‐AMCL and 2‐AMCL GRAPPA lines, up to a maximum of 12 single (1‐AMCL) lines or 6 pairs (2‐AMCL) of lines. ROI, region of interest

Parallel imaging performance of the 10‐channel neck coil is assessed in Figure 2. For comparison, retained SNR maps (1/g) for R = 2 and R = 3 GRAPPA accelerated acquisitions are shown on the top row of Figure 2A. On the bottom row, retained SNR maps are shown for reconstructions in which 12 single lines (1‐AMCL) and 6 pairs of lines (2‐AMCL) were estimated. The mean SNR reduction was computed in a region of interest close to the vessel wall for various numbers of 1‐ and 2‐AMCL estimations, and the results are shown in Figure 2B. These results show that the g‐factor is highly dependent on the number of 2‐AMCL estimations, which should therefore be carefully controlled. In this study, a maximum of 12 lines were estimated; therefore, retained SNR was within the upper left triangle of the Figure 2B matrix (minimum ~ 70%). The mean number of corrupted lines in the motion experiments was 8.3 (SD 2.4), which is within the g‐factor–imposed limit of 12 lines. This limit allows correction of approximately 5 to 6 movements during the 4 min acquisition.

**Figure 3:**
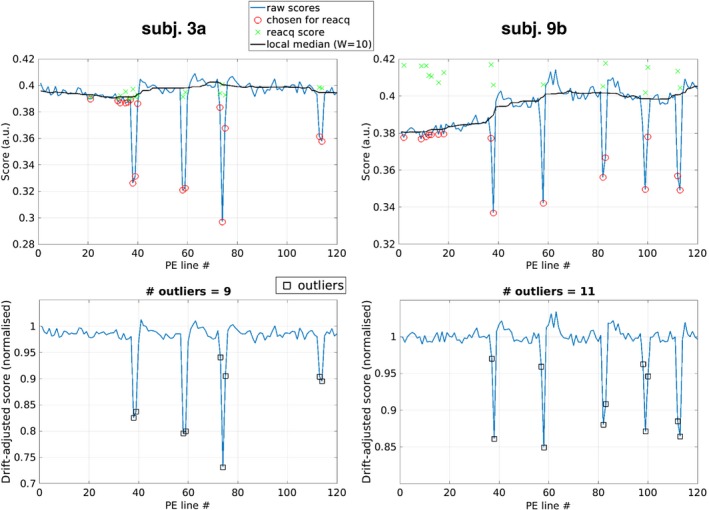
Quality scores for subject scans 3A and 9B. Top row: Scores before drift adjustment, showing lines chosen for reacquisition based on low‐quality scores circled in red and the reacquired scores shown as green crosses. The black line indicates the local median, with a sliding window of width 10 used to remove the drift in quality scores during the scan. Bottom row: Scores after drift adjustment, with black squares indicating which lines were identified as motion‐corrupted (i.e., scores 3 SDs away from the local median using 25 surrounding scores)

Figure 3 demonstrates that intentional swallowing motion by healthy volunteers leads to clear changes (10%‐25%) in navigator signal. Instances of 3 or more adjacent corrupted lines were detected in both subjects. There were 4 such instances in the 18 motion scans; thus, swallowing movements lasting longer than the g‐factor–imposed 2‐AMCL limit (2 TRs, i.e., 4 s) were relatively rare. Furthermore, 1 of the lines was usually close to the normal navigator signal level, for example, beginning descent or returning to normal nonmotion signal but slightly over the detection threshold. The correction strategy is designed for occasional swallowing; thus, motion that lasts longer than 4 to 6 s or that does not return the neck to a similar position will be challenging to correct for the reasons described in the Introduction.

**Figure 4:**
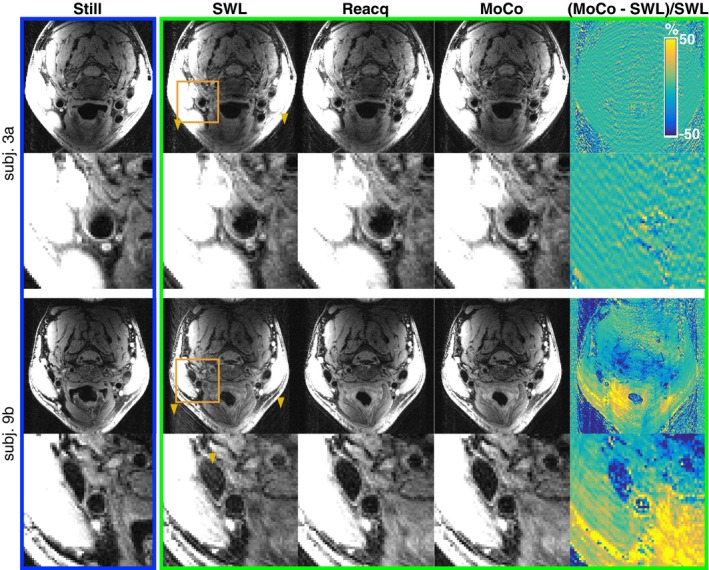
Healthy volunteer images from Still (blue box) to scans with intentional swallowing and SWL, Reacq, and estimation with 2‐AMCL GRAPPA (MoCo). Images are shown for a representative scan (subject 3A) and a scan with central k‐space corruption (subject 9B). The orange box marks the region in the zoomed insets, and the yellow arrows show background ghosting artifacts. MoCo, motion correction with 2‐AMCL GRAPPA; Reacq, replacement of corrupted data with with reacquisitions; Still, motion‐free, gold‐standard scans; SWL, the scans with swallowing motion corruption and the original image reconstruction

Figure 4 compares motion‐free, gold standard scans (blue box) to scans with intentional swallowing, and the various reconstructions (green box) for a representative subject (3A) and the case where the center of k‐space was corrupted by motion (subject 9B). For subject 3A, ghosting is reduced with MoCo relative to SWL and Reacq; however, the vessel is relatively unaffected, as demonstrated in the zoomed insets and the normalized difference map. In the example of central k‐space motion corruption (subject 9b), ghosting and artifacts in the vessel lumen are substantially reduced. The corresponding quality scores for these 2 scans affected by swallowing are provided in Figure 3.

**Figure 5:**
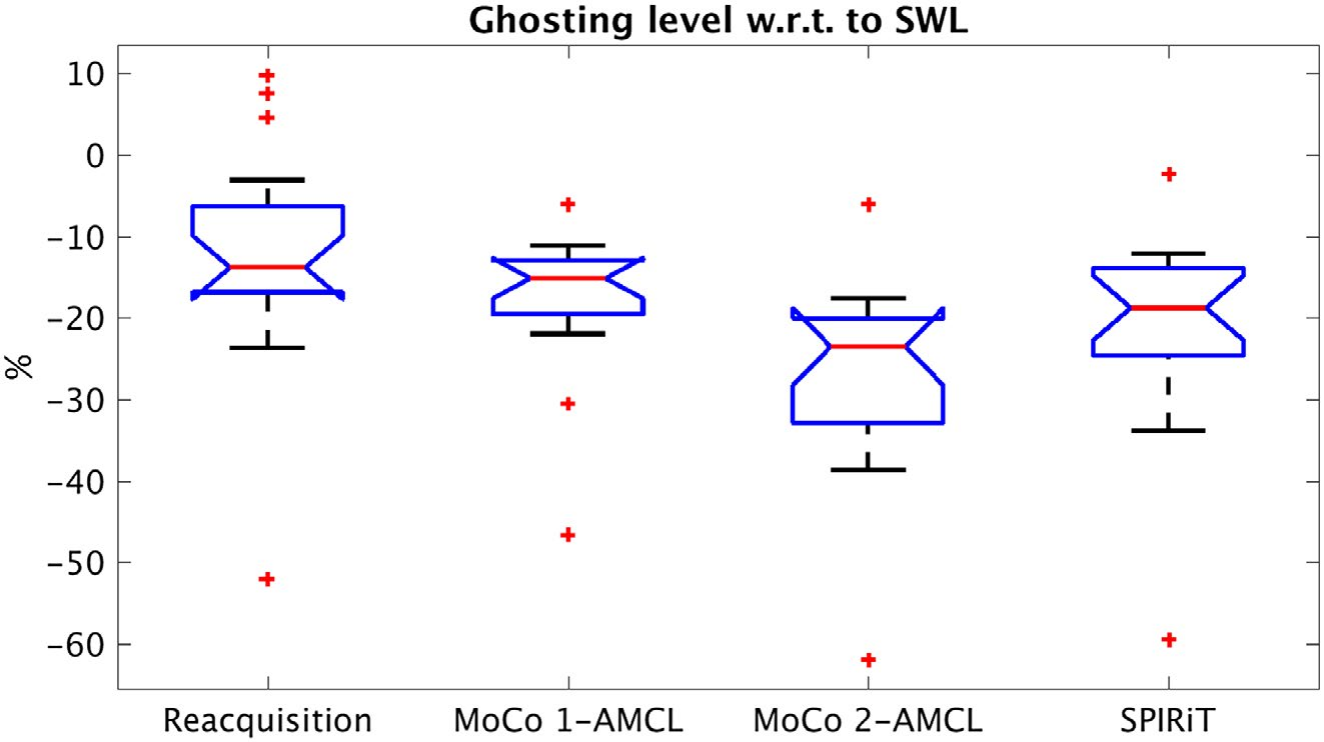
Summary of background ghosting levels in all motion scans normalized to the ghosting level in the original reconstruction. In each scan, the motion‐corrupted lines were replaced with reacquisitions or estimated using GRAPPA or SPIRiT (after replacement of any corrupted central k‐space data with reacquisitions). The boxplots show the median as a red line and the 25th (Q1) and 75th (Q3) percentiles as the boundaries of the blue box. Outliers are defined to be larger than Q3 + (Q3‐Q1) or smaller than Q1 − (Q3‐Q1) and are plotted as red crosses. The black whiskers extend to the most extreme data value that is not an outlier (i.e., up to a maximum of (Q3‐Q1) away from the blue box). The notches on the blue box represent the 95% confidence level on the median; therefore, nonoverlapping notches indicate significantly different median values. SPIRiT, iterative self‐consistent parallel imaging reconstruction

A summary of the ghosting reduction in reconstructions of all scans (9 subjects, 2 scans each) is shown in Figure 5. 2‐AMCL GRAPPA reconstruction has a median ghosting reduction of 24% compared to the uncorrected image and is significantly improved (*P* < .05) relative to reacquisition and 1‐AMCL GRAPPA. The outliers with > 40% reduction are the subject 9B case for which the k‐space center was corrupted by motion. Note that reacquisition image quality is variable and sometimes increases ghosting compared to the original uncorrected image. This is likely to be caused by the problem of inserting inconsistent reacquired data acquired at the end of the scan, by which time the subject may have changed position slightly. GRAPPA (with 2‐AMCL reconstructions) reduces ghosting in all cases and has marginally less ghosting than the SPIRiT reconstruction.

In the 9 healthy volunteers (subject 1‐9A), carotid wall/lumen CNR showed a small but significant improvement of 3% (*P* < .05) (Supporting Information Figure S4) between MoCo and SWL. Vessel sharpness (measured by image edge profile acutance) of Still, SWL, and MoCo data (2‐AMCL GRAPPA) were not significantly different, as shown in Supporting Information Table S1. Carotid T_2_ values of all 9 volunteers (subject 1‐9A) estimated from SWL data were significantly different from those estimated from Still or MoCo, whereas T_2_ differences between Still and MoCo were not significant (Supporting Information Table S1). If the center of SWL k‐space was corrupted by swallowing motion (subject 9b), the MoCo data increased wall/lumen CNR by 14% (*P* < .05) (Supporting Information Figure S5) and image edge profile acutance by 6% (*P* < .05) (Supporting Information Table S1), and it significantly reduced carotid T_2_ values (Supporting Information Table S1) (Supporting Information Figure S5).

**Figure 6:**
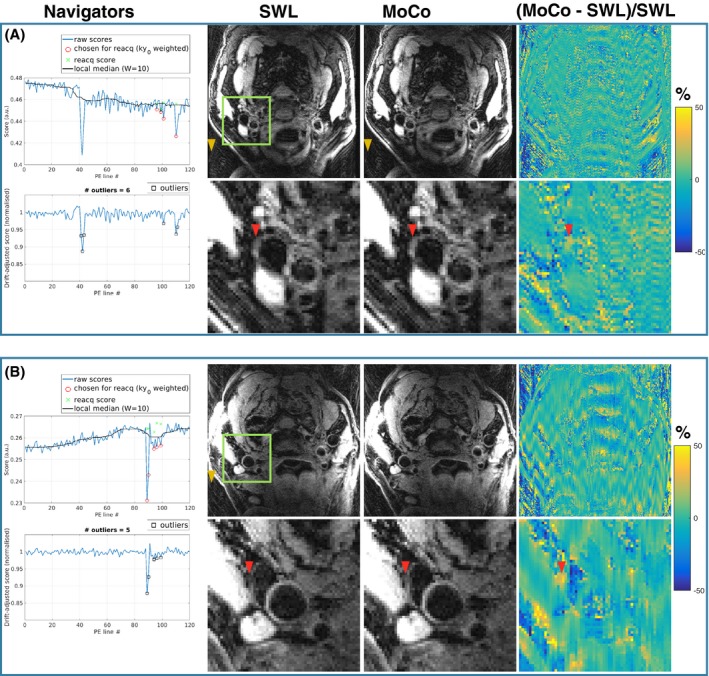
Navigator motion information and images from 2 patient scans where image quality improved. The navigators show that data close to the center of k‐space was corrupted by motion. Comparison of the original reconstruction, estimation with 2‐AMCL GRAPPA (MoCo), and the normalized difference map shows reduced ghosting (yellow arrows) and recovered signal (red arrows). The green box marks the region in the zoomed insets

Figure 6 shows results from scans of 2 patients with carotid atherosclerosis in which image quality was improved. The navigator information demonstrates that clinical scans can have periods of brief motion, and that in these 2 cases there was motion corruption close to the center of k‐space. The MoCo images have reduced background ghosting, and signal was recovered in the vessel wall, as shown in the zoomed insets and normalized difference maps.

**Figure 7:**
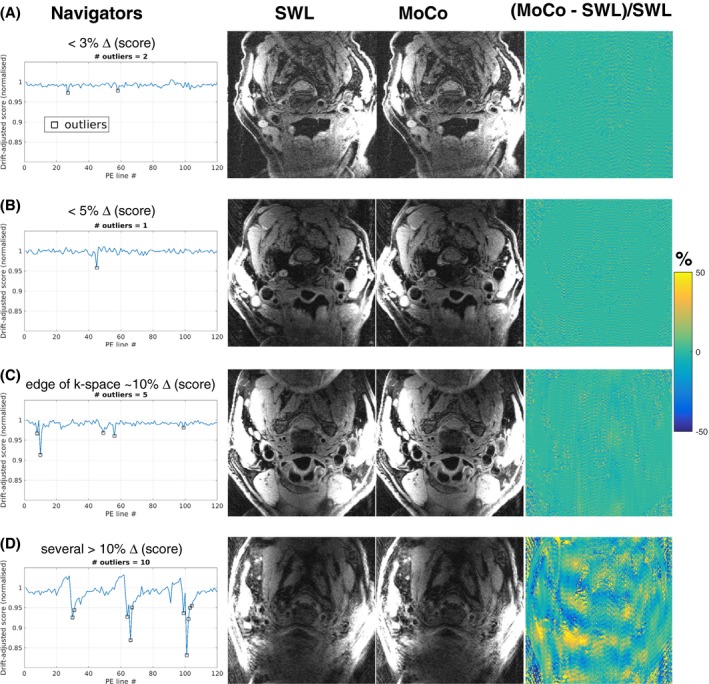
Examples of navigator motion information and the corresponding image quality in scans of 4 patients with carotid atherosclerosis. In some scans, there was low level of motion and only small changes in navigator score (rows A and B), which resulted in low levels of artefact in both the original image reconstruction and the corrected images (MoCo). Larger changes in navigator score further from the edge of k‐space (row C) also result in low levels of artifact. Repeated substantial motion (row D) caused severe artifacts that could not be adequately corrected with MoCo

Figure 7 shows how the navigators provide useful motion information that is relevant to image quality and, further, whether MoCo reconstruction will improve quality. In cases of small changes in the navigator score (Figure 7A,B) or larger changes in score at the edge k‐space (Figure 7C), the original images have low levels of artifact. Scans with several movements and large changes in navigator score have severe artifacts that cannot be adequately corrected with MoCo.

**Figure 8:**
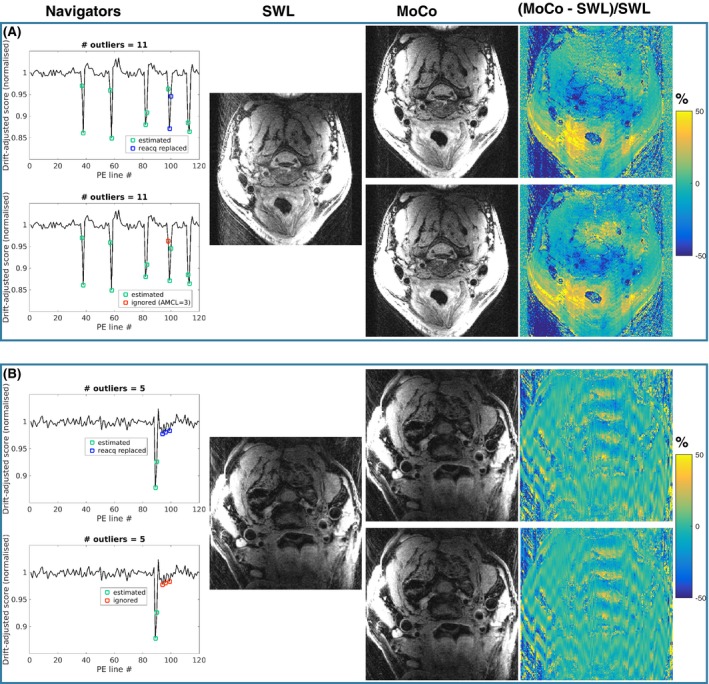
Comparison of different approaches for dealing with motion‐corrupted data at the center of k‐space. (A) Volunteer data where replacement of central k‐space data with reacquisitions (upper row) resulted in similar image quality to the reconstruction with data estimation (lower row). In the estimation reconstruction, estimation of all three adjacent motion‐corrupted lines would require 3‐AMCL estimation, which causes substantial g‐factor noise amplification; thus, the line with the least motion corruption (highest score) was ignored. (B) Patient data where there were outliers on either side of the center of k‐space; therefore, it was not possible calibrate a GRAPPA kernel from a sufficiently wide central k‐space section that included the central k‐space line. In this case, the motion corruption of those lines was minor; thus, the reconstruction ignoring those outliers was of similar quality

Figure 8A shows that estimating the central k‐space data is possible instead of replacing them with reacquisitions and that the image quality is similar. In the example of Figure 8B, there were outliers on either side of the center of k‐space; thus, it was not possible to calibrate a GRAPPA kernel from a sufficiently wide central k‐space section that included the central k‐space line. In this case, the motion corruption of those lines was minor such that the reconstruction ignoring those outliers was of similar quality. However, in the potential case of large motion in this region, the GRAPPA calibration could be of poor quality. The small period of reacquisition weighted toward the center of k‐space is intended to provide robustness to this situation. An additional comparison of reacquisition versus estimation of central k‐space data is provided in Supporting Information Figure S6.

**Figure 9:**
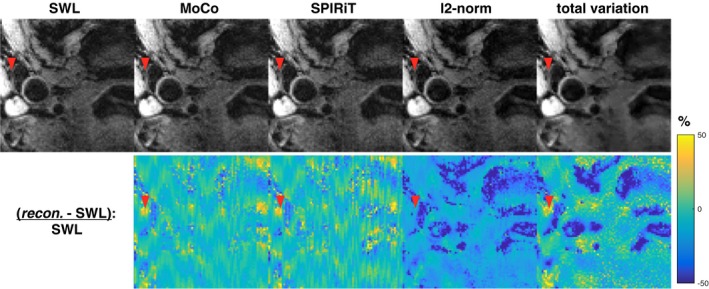
Comparison of estimation approaches with parallel imaging, MoCo (GRAPPA), and SPIRiT, with combined iterative reconstructions using l2‐norm and total variation regularization. No reacquisitions were used in any of the reconstructions

**Figure 10:**
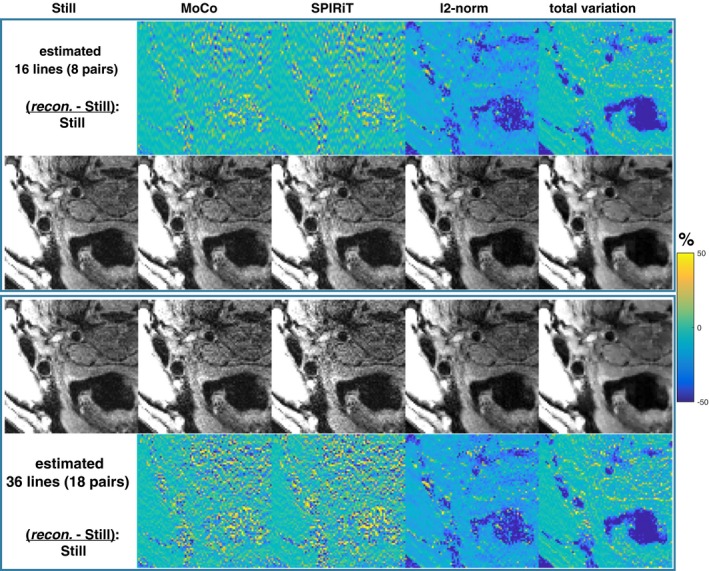
A Still healthy volunteer scan was used to compare performance of image reconstructions with high undersampling factors. 16 and 36 missing lines were estimated. The lines were grouped in pairs; thus, there were 8 and 18 2‐AMCL pairs

Figure 9 shows that the SPIRiT reconstruction has similar performance to MoCo (GRAPPA) in the patient data shown in Figure 6B. The regularized iterative reconstructions suppress noise, although the contrast is altered. Regularization with l2‐norm does not recover the signal in the vessel region, and total variation regularization flattens contrast within structures. However, the iterative reconstructions are more robust to high levels of undersampling, as shown in Figure 10, where 16 and 36 missing lines were reconstructed in a Still healthy volunteer scan.

## 4 DISCUSSION

The technique presented here included 3 components: a navigator echo to identify motion‐corrupted data, a minimal reacquisition for central k‐space lines, and estimation of remaining motion‐corrupted data with parallel imaging. Estimation showed image quality benefits over using reacquisition alone. Motion corruption was detected quickly using a navigator echo (rather than multi‐channel information^25^, ^26^, ^27^, ^28^, ^29^), allowing for real‐time decisions on data quality. This allows the possibility to reacquire any data at the center of k‐space that is severely corrupted. The additional 9 ms navigator per echo train did not affect the overall scan time. A short reacquisition period of ~5 lines was used in patient scans with weighting toward central k‐space (e.g., Supporting Information Figure S3) rather than the 16 reacquisitions acquired for testing in the volunteer scans, which reduced the reacquisition period for this sequence to 10 s from 32 s. The option to reacquire the central k‐space lines is valuable due to the high energy of this signal and the potential impact of the errors.

Estimation of data improves image quality relative to reacquisition alone, as shown in Figures 4 through 5. The finding that reacquisition occasionally increased the ghosting level appears to support the hypothesis that inserting inconsistent k‐space data can be detrimental. The improvement with GRAPPA estimation suggests that there could be improved data consistency, which is in agreement with other previously reported techniques.^25^, ^26^, ^27^, ^28^, ^29^ Although background ghosting was reduced by reacquisition and estimation, the effect on the carotid wall (which is thin in healthy volunteers) was not significant unless the k‐space center was corrupted.

The proposed acquisition and reconstruction were shown to improve image quality in 2 patient scans when there were brief motions during acquisition of data close to the center of k‐space (Figure 6). The patient scans showed how the navigator can measure relevant motion information (Figure 7) and that brief movements were relatively common.

The benefits of using GRAPPA estimation are somewhat tempered by the SNR loss caused by excessive 2‐AMCL reconstructions of lines, as shown in Figure 2. The potential SNR variation between different scans (with different amounts of motion) may be undesirable in some settings, but the number of 2‐AMCL reconstructions could be limited to control the range (see Figure 2B). The SNR performance also depends on the specific characteristics of the coil. Note that the g‐factor SNR reduction remained comparable to an R = 2 acceleration (see Figure 2A), which suffers further from substantial SNR reduction due to undersampling half of the *k_y_* lines (60 vs. ~10). An alternative strategy of minimising the scan time to reduce the opportunity for movement with conventional GRAPPA acceleration can be beneficial when there is sufficient SNR to allow for an undersampled acquisition. Reconstruction of corrupted data in an accelerated scan would be more challenging, for example, with R = 2 conventional GRAPPA, motion corruption in 1 line would require estimation of 3 adjacent k‐space lines. As shown in Figures 9 and 10, regularized iterative reconstructions would be less prone to g‐factor effects in such situations, but there would be altered image contrast.

The quality score occasionally drifts during the acquisition and was accounted for with a sliding window median estimation (see Figure 3). This could be due to slower drift movement of the neck, the amplitude of which can be comparable to the slice thickness^10^ because the quality score is proportional to the total signal within the slice. The problem for reacquisition of score variation during the scan is avoided if online scores are weighted toward central k‐space (using the function shown in Supporting Information Figure S3).

The grouping of motion‐corrupted data (i.e., the number of 2‐AMCL estimations) would be reduced with a modified k‐space acquisition scheme. For example, a randomized line ordering, rather than a sequential acquisition through k‐space, could reduce the number of occasions that contiguous k‐space lines were corrupted by motion lasting longer than the 2 s TR period by spreading corrupted data through k‐space. Investigating the impact of phase‐encode ordering will be the subject of future work. Similarly, the k‐space center was acquired toward the end of the scan (line 97 of 120) and perhaps could be acquired earlier to minimize the probability of motion corruption. The proposed reacquisition strategy weighted toward the center is intended to oversample this important region.

An advantage of this study over prior work is that the same data were used for the SWL and MoCo comparison rather than repeated scans with similar motion, allowing for clear assessment of the effect of replacing corrupted data. Although the MESE sequence is itself highly segmented, 3D‐encoded sequences may be more sensitive. Implementing a similar correction for 3D imaging will be the subject of future work.

On the general applicability of the method, the correction was designed for occasional movement of the neck when the patient returns to approximately the same position. Our aim was to explore to what extent artifacts in MESE, thought to be caused by brief motion events, can be corrected by detecting them and reacquiring the data or by estimating based on surrounding data assumed to be consistent. An advantage of this approach is that, if successful, it could be implemented with relatively small modifications to the scan. The results indicate that these appear to be reasonable assumptions for swallowing or coughing motions, and hence the proposed approach can reduce artifacts. However, more severe artifacts appear to be caused by bulk motion for which more advanced strategies, such as motion‐robust acquisition trajectories and prospective slice position corrections, should be considered. Respiratory motion exceeding ~1 mm, primarily in the head–feet direction, has been reported.^10^, ^11^ Furthermore, over 2 mm of drift motion was consistently observed in a study of 19 healthy volunteers,^10^ and artifacts caused by such bulk changes in neck position will not be effectively corrected using reacquisition or estimation of k‐space data. Corrections that address drift, respiration, and bulk motion, which to our knowledge have not been described to date, could be extremely valuable for clinical carotid wall imaging.

SENSE reconstruction^31^ was not investigated here but would also require some calibration of coil sensitivities. However, with simultaneous autocalibrating and k‐space estimation (SAKE) calibration‐free parallel imaging,^48^ the central k‐space reacquisition may not be necessary. In this work, motion detection with reconstruction of corrupted data was developed for the conventional Cartesian MESE acquisition, but further modifications to the acquisition offer interesting possibilities. Compressed sensing^49^ could be applied to modified k‐space trajectories, for example, radial sampling, that could be intrinsically more robust to motion.^50^, ^51^ Using randomized phase‐encoding within an echo train could allow T_2_ mapping from undersampled data.^52^ Recent work on improving parallel imaging in multi‐contrast acquisitions is also relevant to these T_2_ mapping scans.^53^

## 5 CONCLUSION

This study demonstrated that swallowing motion can be detected with a navigator echo at the end of a 2D MESE readout. The corrupted data can be replaced with reacquired data or estimated with parallel imaging to reduce ghosting artifacts caused by swallowing, with median reductions of 14% (Reacq) and 24% (MoCo) in 18 scans with 9 subjects. The lower ghosting with MoCo estimation is potentially due to improved data consistency because reacquired data could be in a different position. In the presence of corruption of central k‐space data, reacquisition and estimation are particularly effective for improving image quality and the resulting vessel CNR, T_2_, and sharpness. Otherwise, small but significant (*P* < .05) changes were observed in CNR and T_2_ but not in sharpness. The SNR reduction when estimating corrupted data was assessed for a 10‐channel coil, and it was found that reconstructing multiple contiguous lines (e.g., pairs of lines) has a substantial effect on g‐factor noise amplification and should therefore be limited.

The scans of 12 patients suggest that the navigator is useful for detecting relevant motion information. Motion correction improved image quality in 2 patient scans when there was motion corruption close to the center of k‐space. Parallel imaging estimation, with modest reacquisition of central k‐space data to ensure that calibration data is available, is expected to be a robust strategy for correction of artifacts caused by brief motion events.

## Supporting information


**FIGURE S1** Example demonstrating the effect of motion‐corrupted central k‐space data on image quality. Severe artifacts can be avoided with reacquisition of these corrupted data. The plot of quality scores shows the lines identified as corrupted as red circles and the reacquired quality scores as green crosses. The *k_y_* = 0 line is line 97
**FIGURE S2** Demonstration of ghosting level assessment. (A) Examples of ghost (blue) and background (red) regions of interest (ROIs) overlaid on an image acquired without intentional swallowing. Ghosting manifests in the anterior‐posterior direction thus the background ROIs are unaffected and provide the signal level that would be achieved by perfect correction (100% reduction in ghosting). (B‐D) Histograms of intensity distributions in the background ROI of the original reconstruction (B) and the ghost ROIs of the original SWL (C) and 2‐AMCL GRAPPA (D) reconstructions. The median of each distribution is indicated by the green line. (E) Median values of the ghost distribution for the range of image reconstructions (blue line) with the median value of the background distribution in the SWL reconstruction (red dotted line). (F) Ghosting reduction relative to the SWL image. These values are calculated from (E) by subtracting the background contribution and then dividing by the SWL ghost level. 100% reduction indicates ghosting at the level of the background noise
**FIGURE S3** Example of the weighting function for quality scores (used in the patient scans) to prioritize central k‐space data during the online reacquisition
**FIGURE S4** Carotid wall CNR mean values ± SD for subjects 1‐9a (9 volunteers × 2 arteries × 5 slices) and, separately, for subject 9b where the centre of k‐space was corrupted by swallowing
**FIGURE S5** Carotid T2 maps from original SWL and motion‐corrected data in the case of swallowing during the acquisition of the k‐space centre (subj. 9b)
**FIGURE S6** Patient data comparing replacement of central k‐space data with reacquisitions (upper row) with data estimation (lower row). In this case, the navigator scores with higher levels of noise than the other scans and the blurred images suggest that there were frequent small motions which neither MoCo reconstruction could fully correct
**TABLE S1** Carotid wall IEPA mean values ± SD for subjs. 1‐9a (9 normal volunteers × 2 arteries × 5 slices × 14 echoes) and subj. 9b (2 arteries × 5 slices × 14 echoes), where the centre of k‐space was corrupted by swallowing
