# Supplementary material for: Navigator‐based reacquisition and estimation of motion‐corrupted data: Application to multi‐echo spin echo for carotid wall MRI
Source: Magn Reson Med. 2019 Nov 7;83(6):2026–41. doi: 10.1002/mrm.28063 (PMC7065122; doi:10.1002/mrm.28063)
Supplement: Supplementary file 1 — FIGURE S1 Example demonstrating the effect of motion‐corrupted central k‐space data on image quality. Severe artifacts can be avoided with reacquisition of these corrupted data. The plot of quality scores shows the lines identified as corrupted as red circles and the reacquired quality scores as green crosses. The ky = 0 line is line 97 FIGURE S2 Demonstration of ghosting level assessment. (A) Examples of ghost (blue) and background (red) regions of interest (ROIs) overlaid on an image acquired without intentional swallowing. Ghosting manifests in the anterior‐posterior direction thus the background ROIs are unaffected and provide the signal level that would be achieved by perfect correction (100% reduction in ghosting). (B‐D) Histograms of intensity distributions in the background ROI of the original reconstruction (B) and the ghost ROIs of the original SWL (C) and 2‐AMCL GRAPPA (D) reconstructions. The median of each distribution is indicated by the green line. (E) Median values of the ghost distribution for the range of image reconstructions (blue line) with the median value of the background distribution in the SWL reconstruction (red dotted line). (F) Ghosting reduction relative to the SWL image. These values are calculated from (E) by subtracting the background contribution and then dividing by the SWL ghost level. 100% reduction indicates ghosting at the level of the background noise FIGURE S3 Example of the weighting function for quality scores (used in the patient scans) to prioritize central k‐space data during the online reacquisition FIGURE S4 Carotid wall CNR mean values ± SD for subjects 1‐9a (9 volunteers × 2 arteries × 5 slices) and, separately, for subject 9b where the centre of k‐space was corrupted by swallowing FIGURE S5 Carotid T2 maps from original SWL and motion‐corrected data in the case of swallowing during the acquisition of the k‐space centre (subj. 9b) FIGURE S6 Patient data comparing replacement of central k‐space data with reacquisition [file MRM-83-2026-s001.pdf]

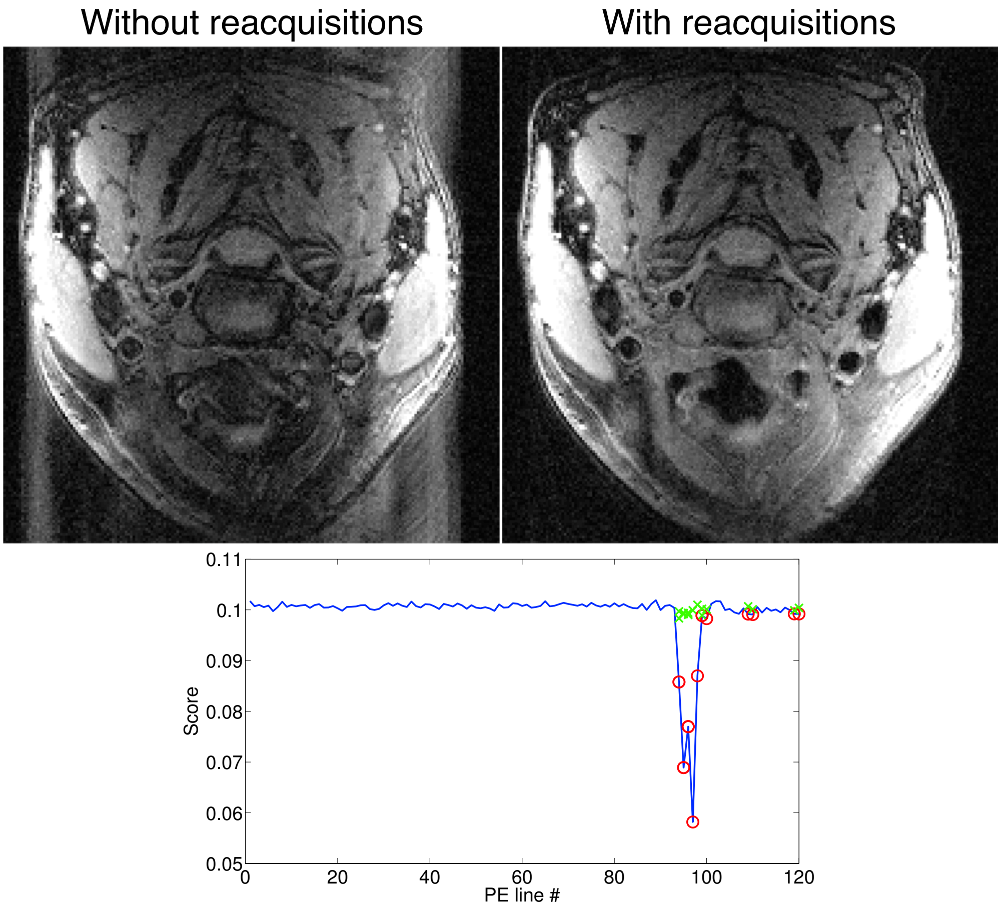

Supporting Information Figure S1:
Example demonstrating the effect of motion-corrupted central k-space data on image quality. Severe artefacts can be avoided with reacquisition of these corrupted data. The plot of quality scores shows the lines identified as corrupted as red circles and the reacquired quality scores as green crosses. The k_y=0 line is line 97.

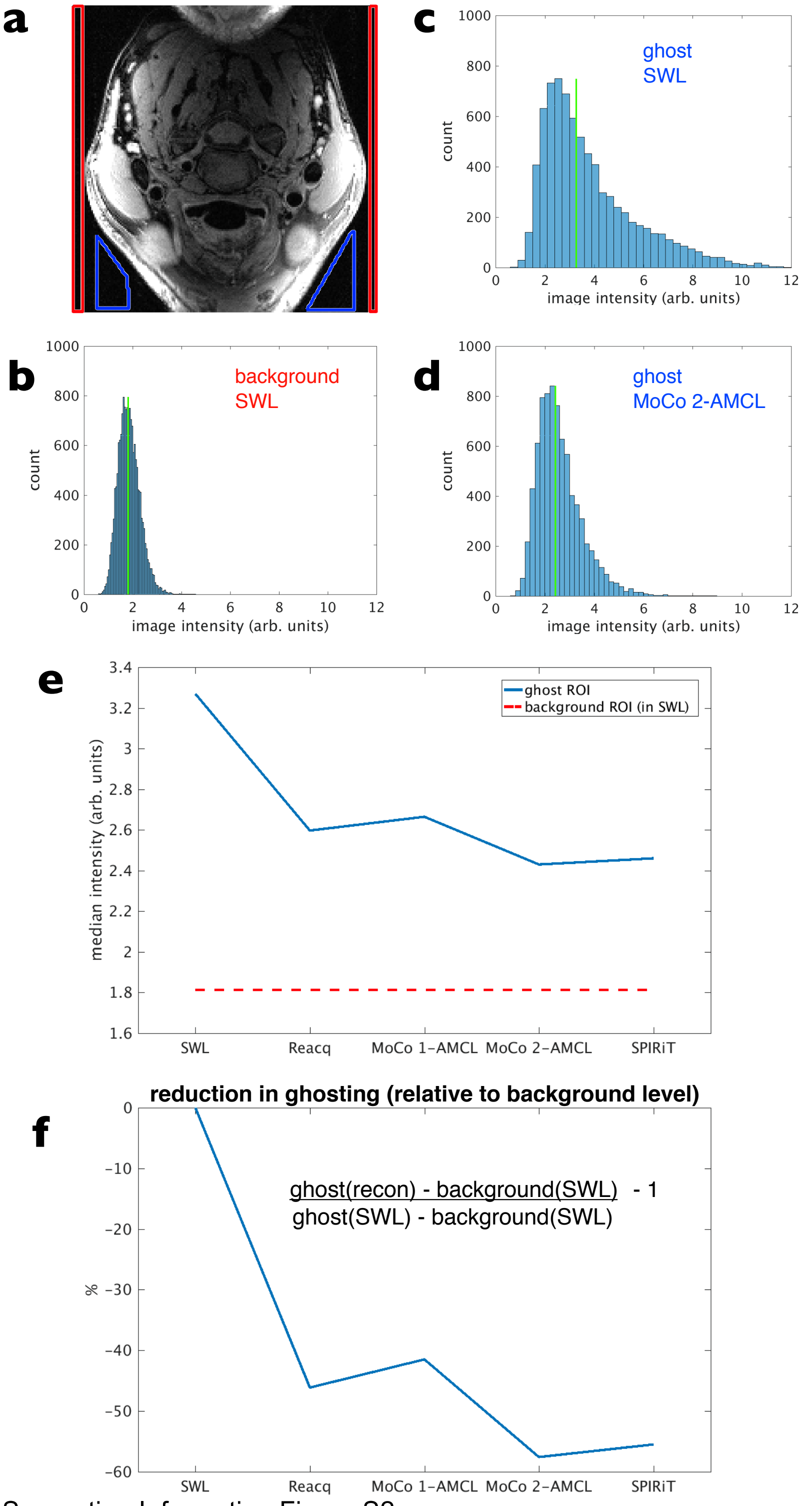

Supporting Information Figure S2:
Demonstration of ghosting level assessment. **(a)** Examples of ghost (blue) and background (red) regions of interest (ROIs) overlaid on an image acquired without intentional swallowing. Ghosting manifests in the anterior-posterior direction thus the background ROIs are unaffected and provide the signal level that would be achieved by perfect correction (100% reduction in ghosting). **(b-d)** Histograms of intensity distributions in the background ROI of the original reconstruction **(b)** and the ghost ROIs of the original SWL **(c)** and 2-ACML GRAPPA **(d)** reconstructions. The median of each distribution is indicated by the green line. **(e)** Median values of the ghost distribution for the range of image reconstructions (blue line) with the median value of the background distribution in the SWL reconstruction (red dotted line). **(f)** Ghosting reduction relative to the SWL image. These values are calculated from (e) by subtracting the background contribution and then dividing by the SWL ghost level. 100% reduction indicates ghosting at the level of the background noise.

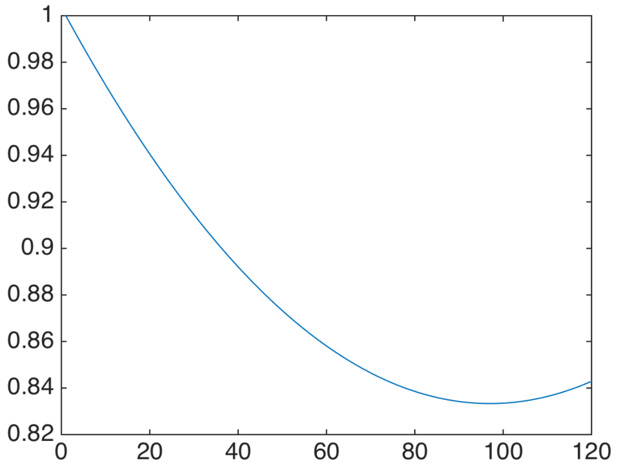

Supporting Information Figure S3:
Example of the weighting function for quality scores (used in the patient scans) to prioritise central k-space data during the online reacquisition.

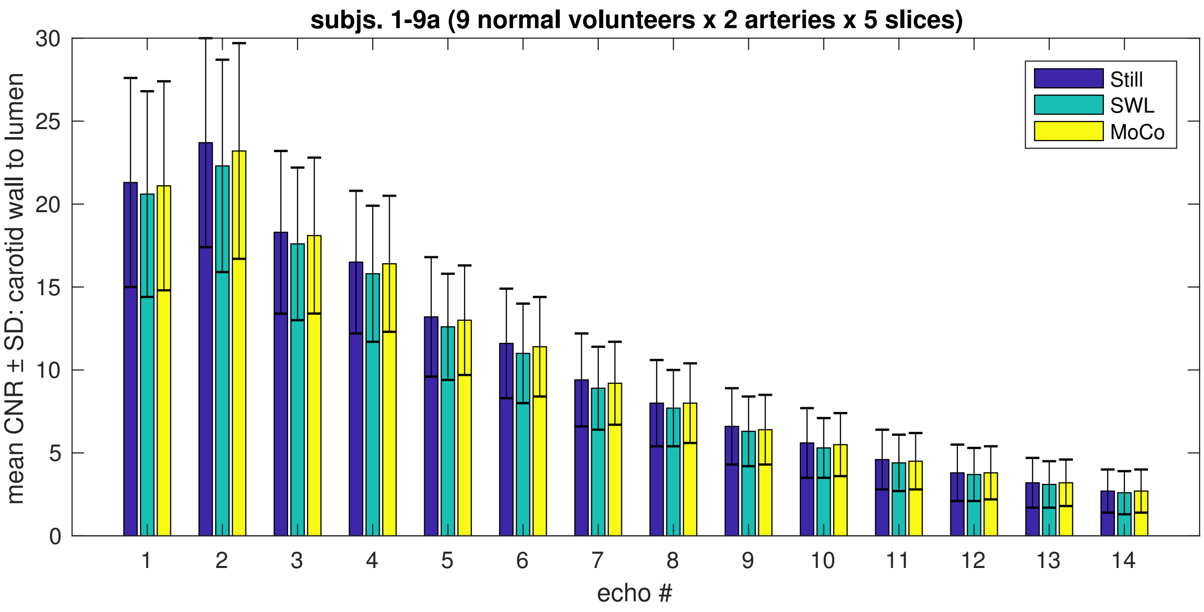

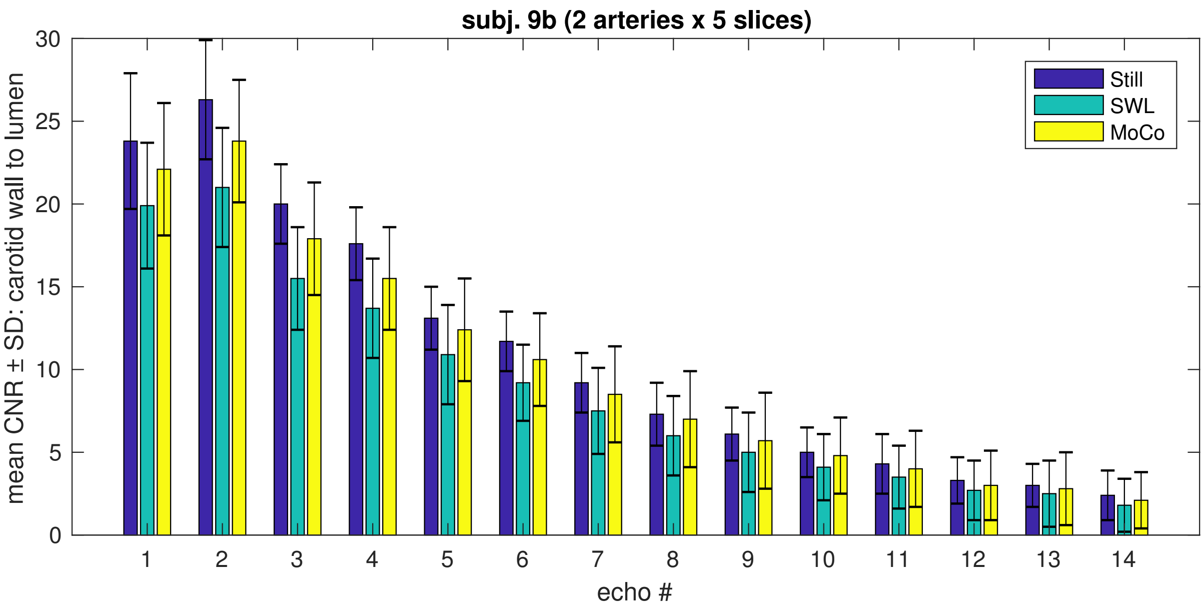

Supporting Information Figure S4:
Carotid wall CNR mean values ± SD for subjects 1-9a (9 volunteers × 2 arteries × 5 slices) and, separately, for subject 9b where the centre of k-space was corrupted by swallowing.

	IEPA × 10^{-4}					
	**Still**	**SWL**	**MoCo**	**Still-SWL**[1]	**SWL-MoCo**[2]	**Still-MoCo**[1]
**NV 1-9a**	561 ± 132	551 ± 149	553 ± 143	ns	ns	ns
**NV 9b***	598 ± 116	560 ± 135	593 ± 120	ns	$P < 0.05$	ns

Carotid wall T2 mean values ± SD for subjs. 1-9a (9 normal volunteers × 2 arteries × 5 slices) and subj. 9b (2 arteries × 5 slices), where the centre of k-space was corrupted by swallowing.

	T2 [ms]					
	**Still**	**SWL**	**MoCo**	**Still-SWL**[1]	**SWL-MoCo**[2]	**Still-MOCO**[1]
**NV 1-9a**	57.9 ± 9.2	59.7 ± 10.8	58.3 ± 10.4	$P < 0.05$	$P < 0.05$	ns
**NV 9b***	52.2 ± 7.9	53.8 ± 7.5	51.6 ± 7.1	$P < 0.05$	$P < 0.05$	ns

*centre of k-space corrupted by swallowing; [1]two-sample t-test; [2]paired t-test

Supporting Information Table S1:
Carotid wall IEPA mean values ± SD for subjs. 1-9a (9 normal volunteers × 2 arteries × 5 slices × 14 echoes) and subj. 9b (2 arteries × 5 slices × 14 echoes), where the centre of k-space was corrupted by swallowing.

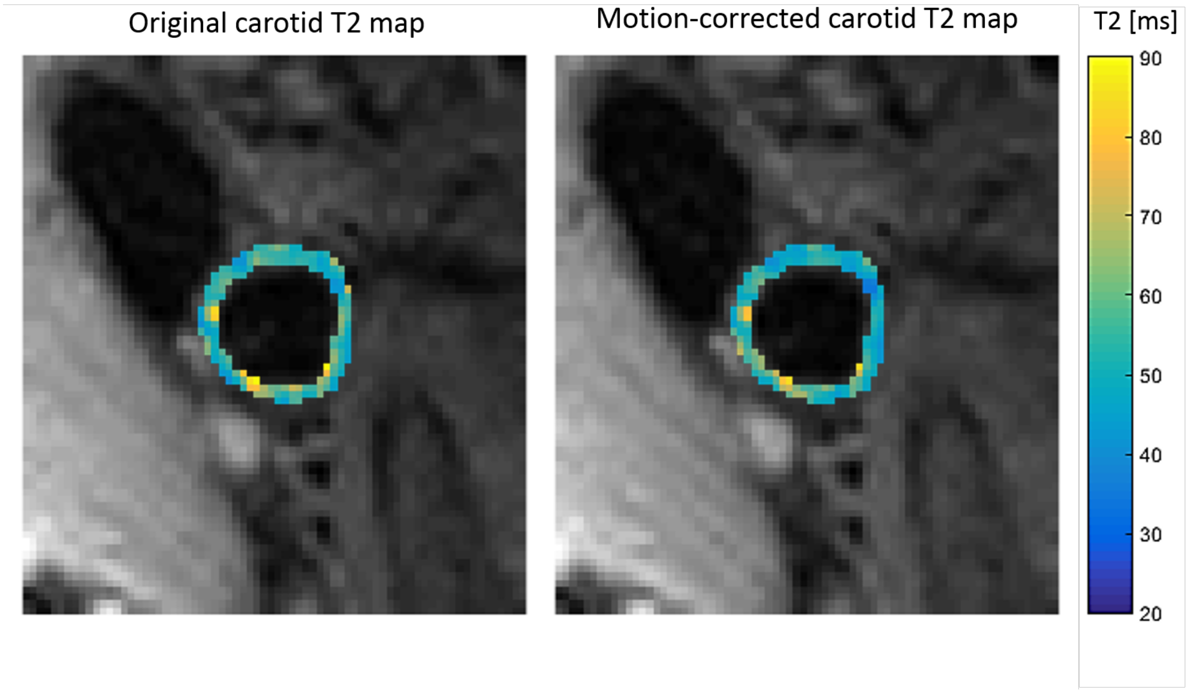

Supporting Information Figure S5:
Carotid T2 maps from original SWL and motion-corrected data in the case of swallowing during the acquisition of the k-space centre (subj. 9b).

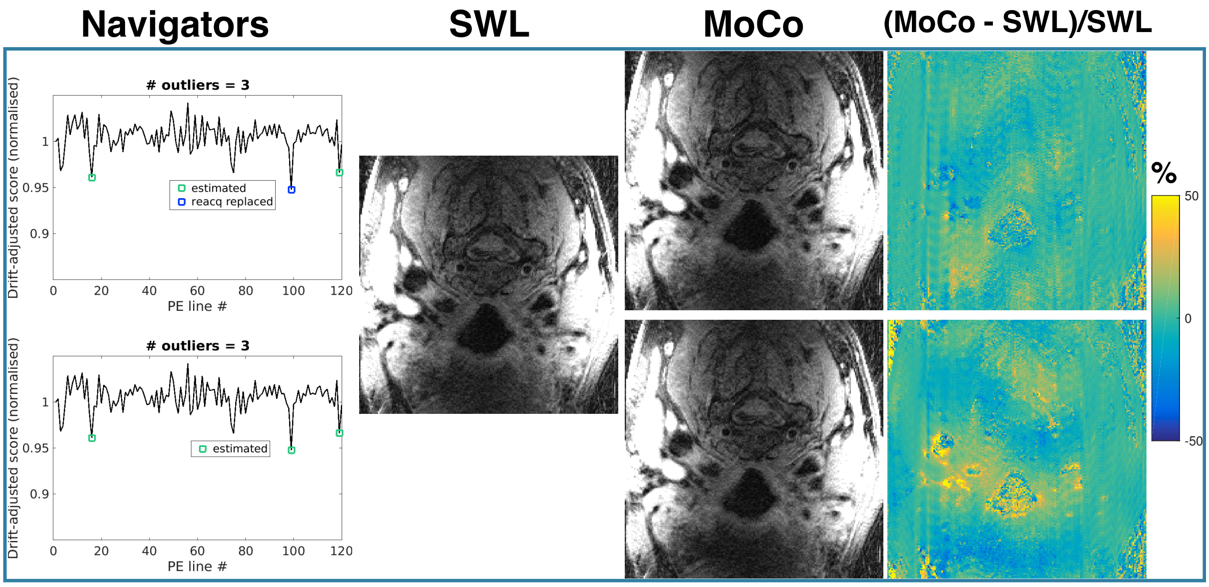

Supporting Information Figure S6:
Patient data comparing replacement of central k-space data with reacquisitions (upper row) with data estimation (lower row). In this case, the navigator scores with higher levels of noise than the other scans and the blurred images suggest that there were frequent small motions which neither MoCo reconstruction could fully correct.
